# Phototropin 1 and 2 Influence Photosynthesis, UV-C Induced Photooxidative Stress Responses, and Cell Death

**DOI:** 10.3390/cells10020200

**Published:** 2021-01-20

**Authors:** Anna Rusaczonek, Weronika Czarnocka, Patrick Willems, Marzena Sujkowska-Rybkowska, Frank Van Breusegem, Stanisław Karpiński

**Affiliations:** 1 Department of Botany, Institute of Biology, Warsaw University of Life Sciences, Nowoursynowska 159, 02-776 Warsaw, Poland; weronika_czarnocka@sggw.edu.pl (W.C.); marzena_sujkowska@sggw.edu.pl (M.S.-R.); 2 Department of Plant Genetics, Breeding and Biotechnology, Institute of Biology, Warsaw University of Life Sciences, Nowoursynowska 159, 02-776 Warsaw, Poland; 3 Department of Plant Biotechnology and Bioinformatics, Ghent University, Technologiepark 71, 9052 Ghent, Belgium; patrick.willems@psb.vib-ugent.be (P.W.); frank.vanbreusegem@psb.vib-ugent.be (F.V.B.); 4 VIB Center of Plant Systems Biology, Technologiepark 71, 9052 Ghent, Belgium

**Keywords:** phototropins, *Arabidopsis thaliana*, chloroplasts, photosynthesis, oxidative stress, transcriptome

## Abstract

Phototropins are plasma membrane-associated photoreceptors of blue light and UV-A/B radiation. The *Arabidopsis thaliana* genome encodes two phototropins, *PHOT1* and *PHOT2*, that mediate phototropism, chloroplast positioning, and stomatal opening. They are well characterized in terms of photomorphogenetic processes, but so far, little was known about their involvement in photosynthesis, oxidative stress responses, and cell death. By analyzing *phot1*, *phot2* single, and *phot1phot2* double mutants, we demonstrated that both phototropins influence the photochemical and non-photochemical reactions, photosynthetic pigments composition, stomata conductance, and water-use efficiency. After oxidative stress caused by UV-C treatment, *phot1* and *phot2* single and double mutants showed a significantly reduced accumulation of H_2_O_2_ and more efficient photosynthetic electron transport compared to the wild type. However, all *phot* mutants exhibited higher levels of cell death four days after UV-C treatment, as well as deregulated gene expression. Taken together, our results reveal that on the one hand, both phot1 and phot2 contribute to the inhibition of UV-C-induced foliar cell death, but on the other hand, they also contribute to the maintenance of foliar H_2_O_2_ levels and optimal intensity of photochemical reactions and non-photochemical quenching after an exposure to UV-C stress. Our data indicate a novel role for phototropins in the condition-dependent optimization of photosynthesis, growth, and water-use efficiency as well as oxidative stress and cell death response after UV-C exposure.

## 1. Introduction

The optimization of light absorption to variable natural conditions is essential to balance photochemistry and photosynthesis reaction rates, and for acclimatory and defense responses in plants. Energy absorbed in excess (excess excitation energy, EEE) is a result of not only high-light exposure, but also UV treatment. EEE causes the damage of Photosystem II (PSII) [1], and it induces photooxidative stress, chloroplast damage, and cell death [2,3,4,5]. Therefore, plants have evolved several avoidance and dissipation mechanisms that protect the photosynthetic apparatus against EEE and optimize photosynthetic reactions [4,5,6,7]. These mechanisms include the chloroplast photorelocation movement, photochemical (qP) and non-photochemical quenching (NPQ), and state transitions [4,5,6,8,9,10].

Chloroplast movement provides yet another protective measure against EEE for maintaining photosynthetic efficiency and is dependent on both phototropin and phytochrome families [6,11]. Phototropins are primary photoreceptors that mediate chloroplast movement in response to blue light and UV-A/B radiation [6,11,12,13]. Arabidopsis possesses two phototropins, phot1 and phot2, that share functional redundancy in processes such as chloroplast accumulation and phototropism control [14], stomata opening [15,16], leaf expansion [17], and leaf positioning [18]. However, they also work independently in some processes. For instance, phot1 is the only phototropin engaged in root phototropism, hypocotyl, and lateral root growth inhibition [19,20,21]. In contrast, phot2 is involved in chloroplast avoidance responses [13,22] and light-dependent nuclear positioning [23].

Both phot1 and phot2 contain two light, oxygen, and voltage (LOV) domains at the protein N-terminus (LOV1 and LOV2) and an additional C-terminal Ser/Thr kinase domain. Both LOV domains are bound with their chromophore, flavin mononucleotide (FMN), which is able to absorb blue (peak around 445–449 nm), UV-A (maximum absorption around 370–380 nm), and UV-B light (around 295 and 315 nm) [24,25]. The photochemical properties of phototropins rely on the interaction between LOV1 and LOV2, which is facilitated by their intervening linker sequence [26]. 

The absorption of light triggers changes in the interaction between LOV2 and the adjacent J_α_ helix [27], which is followed by light-dependent receptor autophosphorylation and the activation of the C-terminal Ser/Thr kinase domain [28], whereas the LOV1 domain modulates the action of LOV2 domain and mediates receptor dimerization [29]. It has been shown that light-dependent autophosphorylation of the serine residues of the kinase activation loop in both phot1 and phot2 is an essential step for phototropin-mediated responses [30,31].

Both Arabidopsis phototropins are expressed in almost all tissues throughout the plant. In leaf tissues, they are localized in epidermal, guard, and mesophyll cells [17,32,33,34]. Despite the lack of transmembrane domains, phot1 and phot2 reside within the plasma membrane, regardless of light conditions [17,35]. Moreover, the plasma membrane and other cellular membranes mentioned below are most probably the sites for phototropin-mediated signaling [36]. Upon blue light irradiation, a fraction of phot1 moves into the cytoplasm [37], while a fraction of phot2 translocates to the Golgi apparatus and *trans*-Golgi network [35]. In addition, some fraction of phot2, and to some extent phot1, has been detected on the chloroplast outer membrane [36]. Light-activated internalization, through a clathrin-dependent endocytic pathway, of phot1 is dependent on the phosphorylation of Ser-851 within the kinase activation loop [26]. Under high blue light treatment, phot1 is ubiquitinated and targeted to the 26S proteasome for degradation, which is a measure to desensitize the receptor [38]. Meanwhile, the light-induced movement of phot2 does not require its phosphorylation and is directed in two separate pathways, one to the Golgi complex or the other to the degradation of the receptor. In fact, in both darkness and blue light, phot2 is continuously degraded and re-synthesized, but in the blue light at the slower rate [39].

Light-regulated chloroplast movement depends on phototropin signaling and predominantly relies on the dynamics of chloroplast actin filaments [40]. In weak light, both phototropins redundantly regulate chloroplast accumulation along periclinal cell walls (perpendicular to the direction of light), which improves the efficiency of light capture [41]. Meanwhile, under EEE conditions, chloroplasts exhibit an avoidance response, which is manifested by positioning at anticlinal cell walls (parallel to the direction of light). This mechanism protect photosystems from photoinhibition and chloroplasts from photodamage [42]. In the *phot1* mutant, an acceleration of both the disappearance and reappearance of chloroplast-actin filaments occurs. Avoidance movements in plants lacking phot1 initiate faster than in wild-type plants [43]. In contrast, the *phot2* mutant exhibits stronger chloroplast accumulation in comparison to the wild type [41], but it demonstrates normal leaf flattening, phototropic response, and stomata opening [14,15,17,33]. *phot2* and *phot1phot2* mutants do not have the ability to reorganize chloroplast-actin filaments, which makes them defective in chloroplast avoidance movements [43].

Although it was shown that phototropins affect photosynthesis through the leaf flattening and leaf positioning [18] as well as stomatal conductance regulation [15] and palisade cell development [33], so far little is known about their influence on the LHCs and PSII efficiency. Since phototropins are important in light perception and chloroplast photorelocation, we assumed that they may also influence light-dependent photosynthetic reactions. In addition, even though the chloroplasts are the major organelles to be affected at the onset of plant cell death [2], so far, the impact of phototropin-dependent chloroplast movements on foliar cell death is weakly described. The only data concerning the cell death regulation by phototropins is that under continuous irradiation with white light, *phot2* mutants exhibit necrotic changes [42,44]. Thus, in the current work, we have examined the influence of phot1 and phot2 on the photosynthetic pigment composition, photochemical and non-photochemical reactions, photooxidative stress response, and cell death. Using transcriptome profiling, we revealed possible signaling pathways that may be phototropin-dependent. As a photooxidative stress inducer, we used UV-C, which has been already well described in causing the damage of PSII [1] and inducing photooxidative stress, chloroplast damage, and cell death in a similar way to EEE [2,3]. Importantly, changes within chloroplasts were shown to be the first observed symptoms of UV-C induced cell death [2]. Therefore, UV-C radiation was used in the current study to explore the influence of phot1 and phot2 on the photooxidative stress response and cell death.

## 2. Materials and Methods

### 2.1. Plant Material and Growth Conditions

*Arabidopsis thaliana* Columbia-0 gl1 (Col-0 gl1) wild-type plants and three mutant lines, *phot1*, *phot2,* and *phot1phot2*, all in the Col-0 gl1 background (the standard Col-0 accession with the glaborous1 mutation that inhibits trichome formation), were used in this study. The seeds were a kind gift of prof. Masamitsu Wada (Tokyo Metropolitan University, Tokyo, Japan). Seeds were stratified for two days and germinated on Jiffy pots. Plants were grown in standard laboratory conditions (8 h photoperiod, PPFD: 80 µmol m^−2^s^−1^), 50% relative air humidity, and temperature day/night: 22/18 °C). Unless specified otherwise, four-week-old-plants were taken for analyses. 

### 2.2. Morphological and Physiological Traits Determination

Rosette size was measured with a FluorCam (Photon System Instruments PSI, Brno, Czech Republic) for 12–24 plants per genotype from at least two independent experiments. The dry weight of whole rosettes was measured after three-day-long desiccation in 105 °C from 12 to 24 plants per genotype from at least two independent experiments. The number of stomata per mm² of leaf area was calculated using imprints of the abaxial side of leaves. Three leaves (6th, 7th, and 8th) were taken for 9 individual plants per genotype from at least two independent experiments. Leaves surfaces were stuck to an adhesive-covered slides using transparent glue (Medical Adhesive Spray, Hollister) and pressed down for 30 sec. Then, the leaf tissues were removed under a gentle stream of tap water, and glued. The lower epidermis was covered with a coverslip. Pictures were taken using a light microscope (Olympus AX70 Provis) and calculated from three frames of each microscopic sample using Olympus-Provis Cell Sens Standard program. Water-use efficiency (WUE) was shown as dry weight per unit of water used (mg of dry weight per mL of water used) for 12–17 plants per genotype from at least two independent experiments. Plants were grown in the 50 mL Falcon tubes filled with a soil-perlite mixture in a 1:1 proportion. To each Falcon tube, 35 mL of water was added. The top of the soil–perlite mixture was covered with an approximately 1 cm thick layer of moisten soil (soil–water in a 1:1 proportion). Seeds were placed inside a 1.5–2 mm wide hole made in the Falcon tube cup. After seeds germination, Falcon tubes were weighed. After 4 weeks of growing, plants were decapitated, Falcon tubes with soil–perlite mixture were weighed (to calculated the water loss), and the dry mass of each plant was measured. Results were presented as previously described [45,46].

### 2.3. Chlorophyll a Fluorescence Parameter Measurements

Chlorophyll *a* fluorescence parameters were measured with a FluorCam (Photon System Instruments PSI, Brno, Czech Republic) using a standard “Quenching” test for 24 plants per genotype per treatment from at least two independent experiments. Chlorophyll fluorescence terminology has been previously described [46,47]. Immediately before the measurements, plants were kept in the dark for 30 min to determine Fo and Fm parameters.

### 2.4. Photosynthetic Pigment Composition Analysis

Whole rosettes were harvested and frozen in liquid nitrogen. Frozen tissue was homogenized in a TissueLyser LT (Qiagen, Germantown, MD, USA) (5 min; 50 rps, 4 °C) with 1 mL of cold acetone (−20 °C) and subjected to further analysis using an HPLC system (Shimadzu, Kyoto, Japan). Pigments were separated on a Synergi 4u MAX-RP 80A 250 × 4.6 column (Phenomenex, Torrance, CA, USA), according to the protocol previously used [2,48] for 9–12 plants per genotype from at least two independent experiments.

### 2.5. Hydrogen Peroxide Levels Determination

The quantitative measurement of hydrogen peroxide (H_2_O_2_) level was spectrophotometrically determined for 9–12 plants per genotype per time point after treatment from at least two independent experiments, as previously described [2,48]. Visualization of hydrogen peroxide level was done by immersing separate leaves in staining solution containing 0.1% (*m*/*v*) 3,3’diaminobenzidine (DAB) (Sigma-Aldrich), 2 mM dimethyl sulfoxide (DMSO), and 0.05% Tween 20 in Milli-Q water pH 3.8 for 24 h in room temperature [49]. After staining, the leaves were decolorated for 24 h in 0.25% (*m*/*v*) chloral hydrate and observed with Leica M165 FC binocular. The images were saved as .jpg files and if necessary adjusted using Photoshop CS 8.0 software by non-destructive tools (contrast and/or levels) throughout the whole area of an image.

### 2.6. Protein Extraction and Enzyme Activity Measurements

Protein extraction from 100 mg of frozen rosettes and the measurement of superoxide dismutase (SOD), catalase (CAT), and ascorbate peroxidase (APX) activities were spectrophotometrically determined as previously described [2,48] for 9–12 plants per genotype per time point after treatment from at least two independent experiments.

### 2.7. UV-C Treatment

Whole rosettes of four-week-old-plants grown under an 8 h photoperiod were treated with UV-C radiation. The treatment was performed in a way that ensured each plant to receive the same UV-C dose. A precise amount of UV-C radiation (100 mJ cm^−2^) was applied using a 500 Crosslinker (Hoefer Pharmacia Biotech, San Francisco, CA, USA), equipped with lamps emitting light in the wavelength ranging from 250 to 258 nm (type G8T5, 8W; Sankyo Denki, Hiratsuka, Japan).

### 2.8. Cell Death Analysis by Electrolyte Leakage and Evans Blue Staining

The quantification of cell death was performed by measuring the ion leakage from whole rosettes, as previously described [2,48] for 9–12 plants per genotype per time point after treatment from at least two independent experiments. For the visualization of cell death, leaves were stained with 1% (*m*/*v*) Evans blue and vacuum infiltrated for 30 minutes; next, they were incubated for 8 h at room temperature [50]. After staining, the leaves were washed three times with deionized water and decolorated for two days in 0.25% (*m*/*v*) chloral hydrate. Leaves staining was observed with a binocular (Leica M165 FC). The images were saved as .jpg files, and figures were prepared from images processed by the above-mentioned method.

### 2.9. RNA Extraction and cDNA Synthesis

Whole rosettes were harvested and frozen in liquid nitrogen. The RNA was isolated from three biological replicates, each consisting of at least four rosettes. Total RNA isolation was performed using a GeneMATRIX Universal RNA Purification Kit (EURX, Gdańsk, Poland) with the additional step of on-column DNase I digestion. RNA concentration and purity were tested by the spectrophotometric method with BioSpectrometer (Eppendorf, Hamburg, Germany). The RNA integrity was tested by electrophoretic separation in 1% agarose gel. Equivalent amounts of all RNA samples were used for cDNA synthesis with a High-Capacity cDNA Reverse Transcription Kit (Thermo Fisher Scientific, Waltham, MA, USA).

### 2.10. Relative Gene Expression Measurement by Real-Time qPCR

Real-time qPCR was performed in the 7500 Fast Real-Time PCR System using Power SYBR Green Master Mix (Thermo Fisher Scientific, Waltham, MA, USA). Each reaction was performed in three biological replicates and three technical repeats, using the following cycling program: 95 °C for 10 min (enzyme activation), followed by 40 cycles of denaturation in 95 °C for 15 s and annealing/extension in 60 °C for 60 s. Primers were designed with Primer3 software (Primer3Plus, Free Software Foundation, Inc., Boston, MA, USA), and their sequences are provided in Supplementary Table S1. Genes encoding PROTEIN PHOSPHATASE 2A SUBUNIT A2 (PP2AA2, AT3G25800), 5-FORMYLTETRAHYDROFOLATE CYCLO-LIGASE (5-FCL, AT5G13050) and YELLOW-LEAF-SPECIFIC GENE 8 (YLS8, AT5G08290) were used as reference genes, according to the RefGenes tool incorporated in Genevestigator [51]. The specificity of each primer pair was verified using melting curve analysis. The efficiency of real-time qPCR was calculated using a LinRegPCR tool [52]. Statistical analysis of the results, including the calculation of relative gene expression levels and the significance of the difference between tested samples, was performed using REST2009 [53].

### 2.11. RNA Sequencing and RNA-Seq Analysis

The RNA was isolated from 3-week-old non-treated and UV-C treated plants 30 min after plant exposure to 100 mJ cm^−2^ of UV-C. RNA was isolated from three biological replicates, each consisting of at least four rosettes. Total RNA isolation was performed using a GeneMATRIX Universal RNA Purification Kit (EURX, Gdańsk, Poland) with the additional step of on-column DNase I digestion. RNA concentration and purity were determined spectrophotometrically using the Nanodrop ND-1000 (Nanodrop Technologies), and RNA integrity was assessed using a BioAnalyzer 2100 (Agilent). Per sample, an amount of 1000 ng of total RNA was used as input. Using the Illumina TruSeq® Stranded mRNA Sample Prep Kit (protocol version: Document # 1000000040498 v00–October 2017), poly-A containing mRNA molecules were purified from the total RNA input using poly-T oligo-attached magnetic beads. In a reverse transcription reaction using random primers, RNA was converted into first-strand cDNA and subsequently converted into double-stranded cDNA in a second-strand cDNA synthesis reaction using DNA PolymeraseI and RNAse H. The cDNA fragments were extended with a single ’A’ base to the 3’ ends of the blunt-ended cDNA fragments after which multiple indexing adapters were ligated, introducing different barcodes for each sample. Finally, enrichment PCR was carried out to enrich those DNA fragments that have adapter molecules on both ends and to amplify the amount of DNA in the library. Sequence libraries of each sample were equimolarly pooled and sequenced on Illumina HiSeq 4000 (Paired-end kit, 76 cycles, Dual Index, 4 lanes) at the VIB Nucleomics Core (www.nucleomics.be). Reads were aligned to the Arabidopsis genome by STAR (v2.5.2b) [54] using the Araport11 annotation [55]. The number of reads per gene was quantified with the featureCounts function as implemented in the Subread package v1.6.2 [56]. Only protein-coding genes quantified by at least 5 reads in at least six samples (20,086 genes) were retained for downstream differential gene expression analysis using the software package edgeR [57] in R (v3.4.1). Trimmed mean of M values (TMM) normalization [58] was applied using the calcNormFactors function. Variability in the dataset was assessed with a multidimensional scaling (MDS) plot, showing clear separation according to genotype and UV-C treatment. To test user-defined hypotheses, a no-intercept single-factor model was defined combining genotype and treatment factors, e.g., such as *phot1*_UV. Dispersions were estimated with the estimateGLMRobustDisp function. A negative binomial regression model was used to model the overdispersed counts for each gene separately with fixed values for the dispersion parameter as outlined [59] and as implemented in the function glmFit using the above described model. Hypothesis testing was based on likelihood ratio tests. Contrasts of interest were the response between different genotypes under control conditions, the effect of UV stress in each genotype, and the interaction effect of UV stress and genotype. False discovery rate adjustments of the P values were performed with the method described by [60]. The gene expression data were deposited in Gene Expression Omnibus (GEO; http://www.ncbi.nlm.nih.gov/geo/) under accession number GSE143760. Transcripts with significantly altered expression level (false discovery rate (FDR) < 0.01; logFC > |1|), in comparison to the wild type were taken for Gene Ontology (GO) enrichment and functional analysis. Gene ontology enrichment analysis was performed using the ThaleMine tool (v4.1.2-20200127) within the Araport portal [61]. Functional analysis of deregulated transcripts was performed using the MapMan tool [62].

## 3. Results

### 3.1. phot1 and phot2 Have an Impact on Rosette Size, Plant Dry Mass, Stomata Density, and Water Use Efficiency

In order to elucidate the role of phot1 and phot2 on the regulation of morphological traits, such as plant size and biomass, stomata density, and WUE, we used phototropin single *phot1* and *phot2*, and double *phot1phot2* mutants in Col-0 gl1 background along with wild-type Col-0 gl1. 

**Figure 1:**
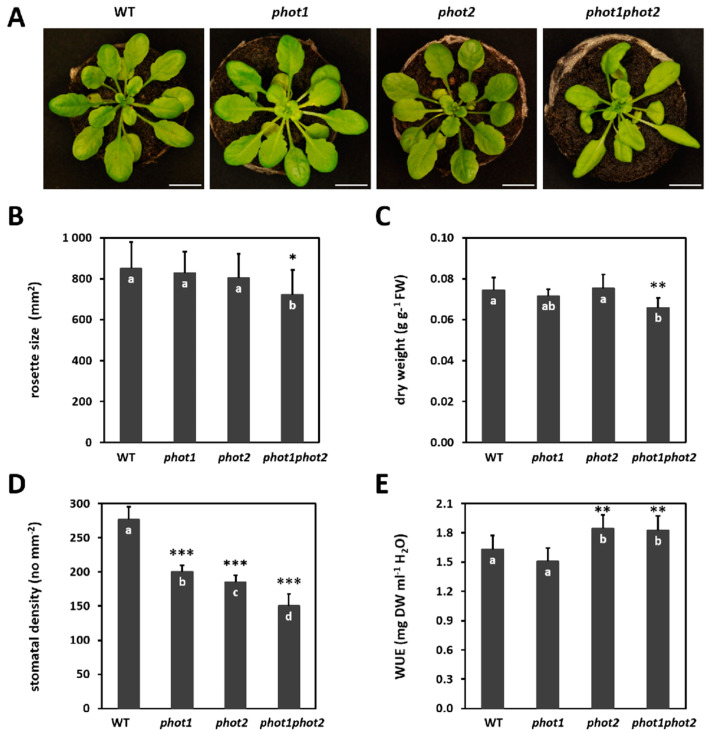
Morphological and physiological traits in 4-week-old *Arabidopsis* wild-type (WT) and phototropin mutants (*phot1*, *phot2*, and *phot1phot2*), in the Col-gl1 background. (**A**) Rosette morphology. Scale bar, 10 mm; (**B**) Rosette size. Values are means (±SD) of 12–24 plants per genotype from at least two independent experiments (*n* = 12–24); (**C**) Dry weight. Values are means (±SD) of 12–17 plants per genotype from at least two independent experiments (*n* = 12–17); (**D**) Stomatal density. Values are means (±SD) of 27 leaf fragments per genotype from at least two independent experiments (*n* = 27); and (**E**) Water-use efficiency (WUE). Values are means (±SD) of 12–17 plants per genotype from at least two independent experiments (*n* = 12–17). Asterisks indicate significant differences from the wild type, according to the Tukey honest significant difference (HSD) test at the level of *p* < 0.05 (*), *p* < 0.005 (**), or *p* < 0.001 (***). Different letters indicate a significant difference at *p* < 0.05 (Tukey’s test).

While *phot1* and *phot2* single mutants, grown in short day and under 80 µmol m^−2^s^−1^ photosynthetic photon flux density conditions, did not differ from the wild type in terms of rosette morphology, the *phot1phot2* double mutant showed curled leaves and a significantly smaller rosette size (Figure 1A,B). Moreover, *phot1phot2* double mutant plants had a reduced rosette dry weight (Figure 1C) and almost half the number of stomata per mm^2^ of leaf blade compared to the wild type (Figure 1D). Significantly decreased stomatal density was also observed for *phot1* and *phot2* single mutants, by 27% and 33%, respectively (Figure 1D). In addition, the *phot2* and *phot1phot2* mutants demonstrated a significantly higher water-use efficiency (WUE) parameter (Figure 1E), which was measured as dry weight per water used.

These results indicate that phot2 together with phot1 has a positive impact on plant biomass production and stomatal density, and thus influences WUE.

### 3.2. phot1 and phot2 Influence Photosynthetic Parameters and Pigment Composition

**Figure 2:**
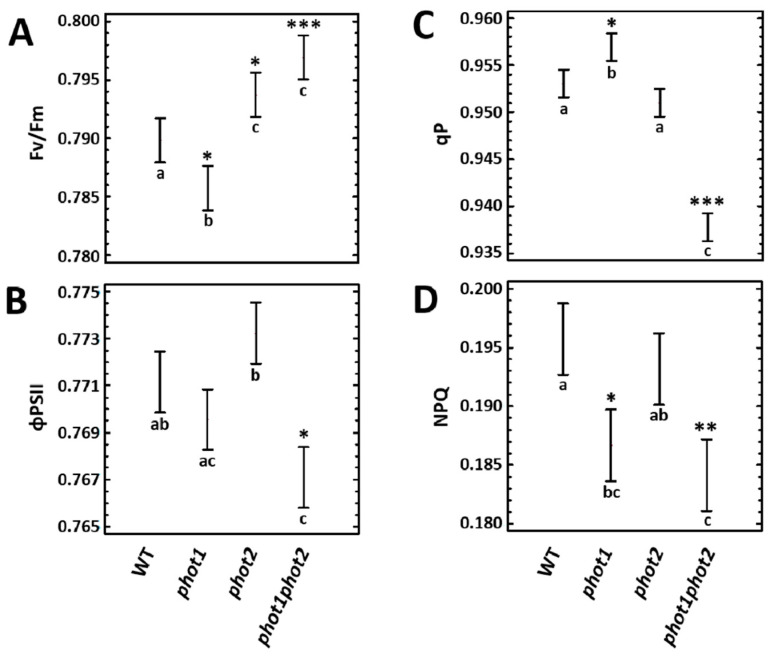
Chlorophyll *a* fluorescence parameters measured in 4-week-old wild-type and mutant plants. (**A**) *Fv/Fm*, maximum quantum efficiency of PSII; (**B**) ΦPSII, quantum yield of PSII; (**C**) *q*P, photochemical quenching; (**D**) NPQ, non-photochemical quenching. Values are means (±SD) of 24 plants per genotype from at least two independent experiments (*n* = 24). Asterisks indicate significant differences from the wild type, according to the Tukey’s HSD multiple comparison test at the level of *p* < 0.05 (*), *p* < 0.005 (**), or *p* < 0.001 (***).

Chlorophyll *a* fluorescence measurements demonstrated that the maximum quantum efficiency of PSII (*F*v/*F*m) was significantly decreased in *phot1* mutant, while it increased in *phot2* and *phot1phot2* mutants compared to the wild type (Figure 2A). However, the operational PSII efficiency (ΦPSII) in *phot1* and *phot2* was similar to the wild type. Meanwhile, the *phot1phot2* double mutant, despite its elevated *F*v/*F*m, showed significantly lower ΦPSII (Figure 2B). However, the difference from the wild type was less than 10%. Taking into consideration the photochemical quenching (*q*P) parameter, which approximates the proportion of PSII reaction centers that are open, *phot1* mutant demonstrated higher *q*P (Figure 2C). In contrast, *phot1phot2* double mutant had a reduced value of *q*P (Figure 2C), which may explain the lower observed ΦPSII (Figure 2B). Lastly, the rate of EEE dissipation through the NPQ reactions was reduced in *phot1* and *phot1phot2* (Figure 2D).

Since we observed changes in chlorophyll *a* fluorescence parameters in *phot1*, *phot2* single, and *phot1phot2* double mutants, in comparison to the wild type, the next step was to evaluate if these differences are caused by the deregulation of the photosynthetic pigment composition. Total chlorophyll content showed to be significantly lower in *phot2* and *phot1phot2* mutants (Table 1). These mutants had decreased levels of both chlorophyll *a* and *b*. The chlorophyll *a* to *b* ratio (chl *a/b*) was significantly increased in all phototropin mutants. Taking into account the carotenoids content, all tested mutants demonstrated significantly lower content of lutein (Table 1) in comparison to the wild-type plants. The de-epoxidation state of carotenoids engaged in xanthophyll cycle (VAZ cycle) was significantly diminished in all *phot1*, *phot2*, and *phot1phot2*, suggesting lower intensity of NPQ processes in these mutants, compared to the wild-type plants. Only a *phot1phot2* double mutant demonstrated significantly reduced β-carotene content. 

These results indicate that phototropins influence the regulation of photochemical and non-photochemical reactions and have a positive impact on photosynthetic pigments composition.

### 3.3. phot1 and phot2 Affect Foliar H_2_O_2_ Homeostasis in Non-Stress and Oxidative Stress Conditions

UV-C radiation triggers photooxidative stress and cell death in Arabidopsis [2,48]. Therefore, we used UV-C radiation to explore the influence of phot1 and phot2 on the redox homeostasis and cell death progression (Supplementary Figure S1). 

**Figure 3:**
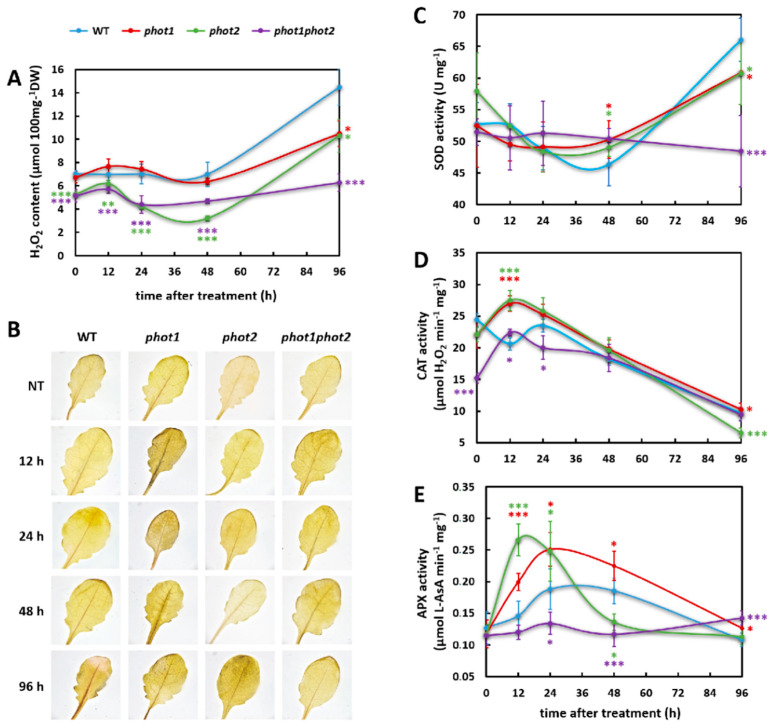
Hydrogen peroxide production and activities of selected antioxidant enzymes in 4-week-old wild-type and mutant plants, determined for non-treated plants and 12, 24, 48, and 96 h after UV-C exposure (100 mJ cm^−2^). (**A**) H_2_O_2_, hydrogen peroxide content; (**B**) 3,3’diaminobenzidine (DAB) staining showing H_2_O_2_ production; (**C**) SOD, superoxide dismutase activity; (**D**) CAT, catalase activity; (E) APX, ascorbate peroxidase activity. Pictures show representative images selected from five different leaves stained per genotype and per time point. Values are means (±SD) of 9–12 plants per genotype and time point from at least two independent experiments (*n* = 9–12). Asterisks indicate significant differences from the wild type, according to the Tukey HSD test at the level of *p* < 0.05 (*), *p* < 0.005 (**), or *p* < 0.001 (***).

Firstly, we assessed the content of hydrogen peroxide (H_2_O_2_) in rosettes before and after a precise dose (100 mJ cm^−2^) of UV-C radiation. In non-treated *phot2* and *phot1phot2*, the level of H_2_O_2_ was significantly lower when compared to the wild type (Figure 3A,B). The same tendency was maintained for these genotypes in all tested time points (12, 24, 48, and 96 h) after UV-C exposure (Figure 3A,B). UV-C stressed *phot1* mutant accumulated significantly less H_2_O_2_, in comparison to the wild type, only 96 h after UV-C exposure.

In a next step, we tested whether altered H_2_O_2_ levels are due to the altered activity of enzymes regulating reactive oxygen species (ROS) homeostasis. Under non-stress conditions, all tested genotypes showed similar activity of superoxide dismutase (SOD) (Figure 3C). Differences among genotypes appeared only 48 h after UV-C treatment, when both *phot1* and *phot2* single mutants demonstrated higher SOD activity in relation to wild type. In contrast, 96 h after UV-C exposure, all phototropin mutants showed significantly reduced activities of SOD when compared to wild-type plants. Catalase (CAT) activity in *phot1phot2* mutants before UV-C exposure was significantly decreased in comparison to the wild type (Figure 3D). Similarly, we observed reduced CAT activity for *phot1* and *phot2* mutants; however, these changes were not statistically significant. Twelve hours after UV-C irradiation, all analyzed mutants showed higher CAT activity, while 24 h after UV-C treatment, the *phot1phot2* double mutant had lower activity of CAT compared to the wild type. Interestingly, all mutants were faster than the wild type in elevating CAT activity after UV-C exposure. The peak of CAT activity for all *phot1*, *phot2*, and *phot1phot2* mutants was 12 h after UV-C treatment, while for the wild-type plants, it was 24 h after UV-C, and even at this time point, the CAT activity did not exceed the initial level present in the wild type under non-stress conditions. Twenty four hours after UV-C treatment, the CAT activity gradually decreased for all tested genotypes. Even though the activity of ascorbate peroxidase (APX) was similar in all non-stressed genotypes, it displayed the most fluctuating activity after UV-C exposure (Figure 3E). In *phot1* and *phot2* mutants, its activity was about two times elevated 12 h after UV-C compared to the wild type. Both single phototropin mutants maintained significantly increased APX activity 24 h after UV-C exposure, while *phot1phot2* demonstrated reduced activity in relation to the wild type. The *phot1* mutant maintained higher APX activity further to 48 h after UV-C stress, while APX activity dropped severely in the *phot2* mutant 48 h after UV-C irradiation, reaching, together with the *phot1phot2* double mutant, a lower level than in the wild type.

All these results indicate that phototropins affect foliar H_2_O_2_ content as well as the fine-tuning of the SOD, CAT, and APX activities after oxidative stress.

### 3.4. phot1 and phot2 Influence the Resistance Toward UV-C Damage

**Figure 4:**
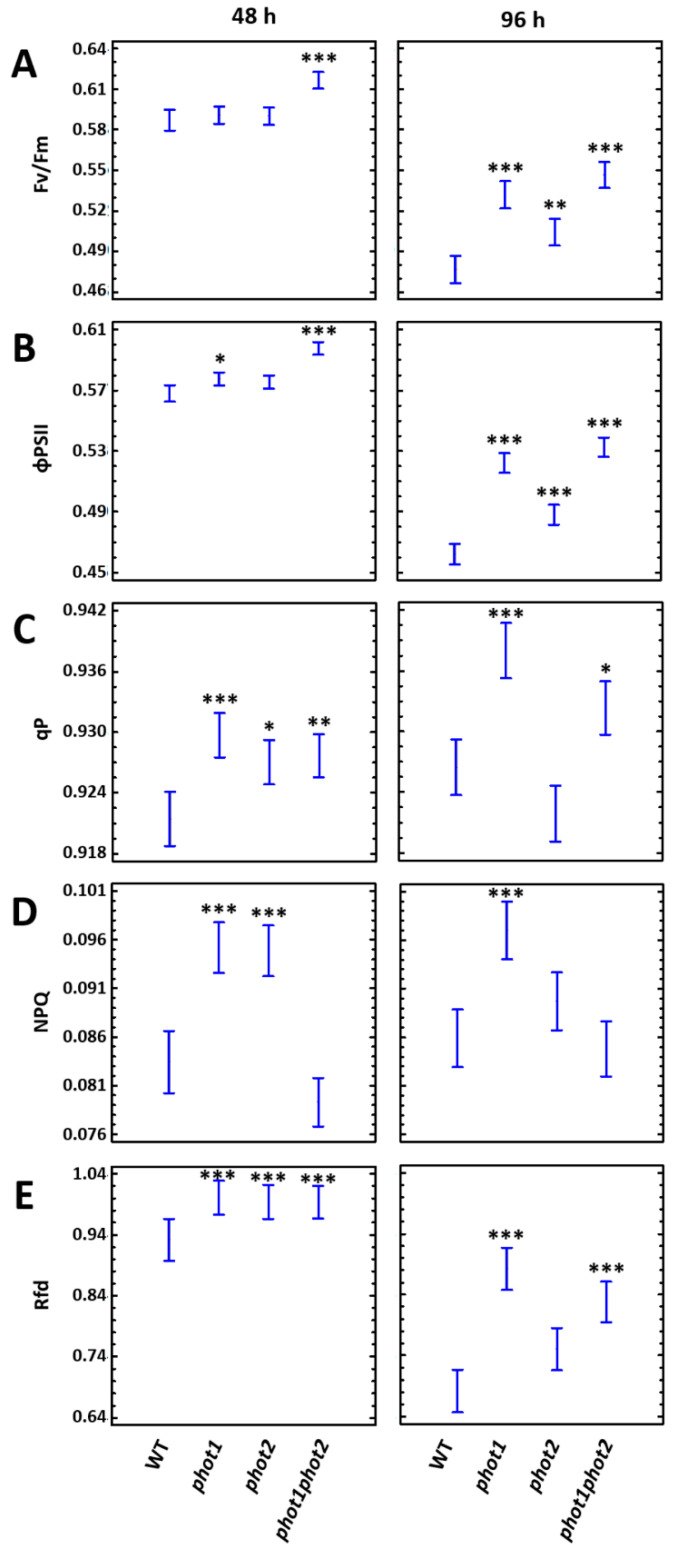
Chlorophyll a fluorescence parameters measured in 4-week-old wild-type and mutant plants before and 48 and 96 h after UV-C exposure (100 mJ cm^−2^). (**A**) Fv/Fm, maximum quantum efficiency of PSII; (**B**) ΦPSII, quantum yield of PSII; (**C**) qP, photochemical quenching; (**D**) NPQ, non-photochemical quenching; (**E**) Rfd, plant vitality parameter. Values are means (±SD) of 24 plants per genotype from at least two independent experiments (*n* = 24). Asterisks indicate significant differences from the wild type, according to the Tukey’s HSD multiple comparison test at the level of *p* < 0.05 (*), *p* < 0.005 (**), or *p* < 0.001 (***).

Since UV-C radiation is known for its negative impact on photosynthetic apparatus [2], and in fact induces EEE [63], our next step was to assess the role of phot1 and phot2 in UV-C triggered changes within the photosynthetic machinery. PSII is the primary target for photodamage and the *F*v/*F*m parameter is a good indicator of photoinhibition [4,5]. In order to examine the susceptibility of the photosynthetic apparatus to photodamage, chlorophyll *a* fluorescence measurements were performed. They indicated that 48 h after UV-C treatment, the *phot1phot2* mutant had significantly higher maximum and operational PSII efficiency, as indicated by the *F*v/*F*m and ΦPSII parameters, respectively, in relation to wild type (Figure 4A,B). With the progression of UV-C-induced damages (96 h after UV-C exposure), all phototropin mutants performed better than the wild type in terms of photosynthetic performance. All *phot1*, *phot2,* and *phot1phot2* had significantly elevated *Fv/Fm* and ΦPSII (Figure 4A,B). Moreover, 48 h after UV-C exposure, all tested phototropin mutants demonstrated significantly elevated *q*P in comparison to the wild-type. This tendency was maintained also in *phot1* and *phot1phot2* 96 h after UV-C irradiation. The NPQ parameter was significantly elevated in *phot1* and *phot2* single mutants 48 h after UV-C treatment and maintained higher in *phot1*, compared to the wild type, also 96 h after stress. Importantly, plant vitality, indicated by the Rfd parameter [64], was higher than in Col-0 gl1 plants in all *phot* mutants both 48 h and 96 h after UV-C stress. 

**Figure 5:**
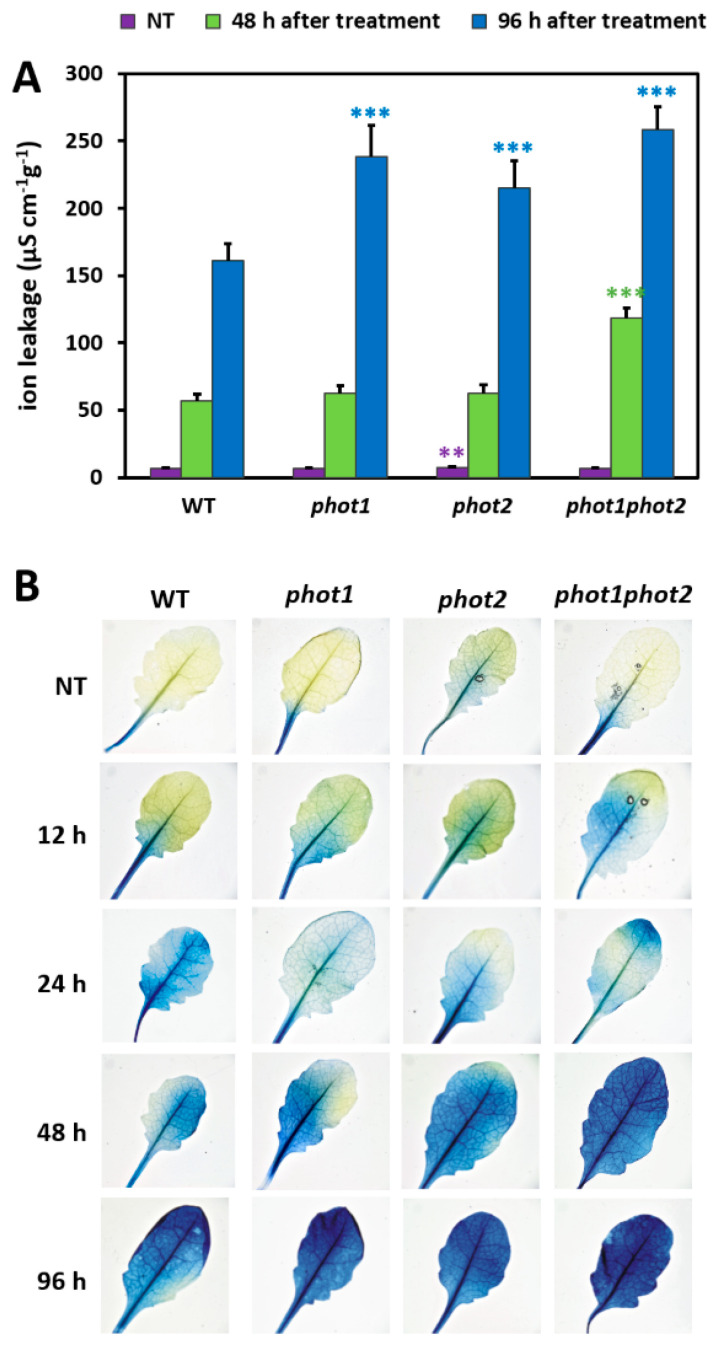
Cell death in 4-week-old wild-type and mutant plants, determined for untreated plants and 48 and 96 h after UV-C exposure (100 mJ cm^−2^). Cell death (**A**) quantified by cellular electrolyte leakage; and (**B**) visualized using Evans blue staining. Pictures show representative images selected from five different leaves stained per genotype and per time point. Values are means (±SD) of 9–12 plants per genotype and time points from at least two independent experiments (*n* = 9–12). Asterisks indicate significant differences from the wild type, according to the Tukey HSD test at the level of *p* < 0.005 (**), or *p* < 0.001 (***).

Genotype-specific changes in the cell death induction were monitored by ion leakage from whole rosettes, as successfully used in our previous works [2,48] and by staining plants with Evans blue. Before UV-C treatment, the ion leakage was relatively low for all tested genotypes, yet the *phot2* mutant demonstrated significantly higher conductivity per fresh weight compared to Col-0 gl1 (Figure 5A,B). Ion leakage assessed 48 h after UV-C irradiation was elevated only in the *phot1phot2* double mutant when compared to the wild type. However, all tested *phot* mutants demonstrated more pronounced cell death, which was quantified 96 h after UV-C exposure, by cellular electrolyte leakage and visualized by Evans blue staining.

**Figure 6:**
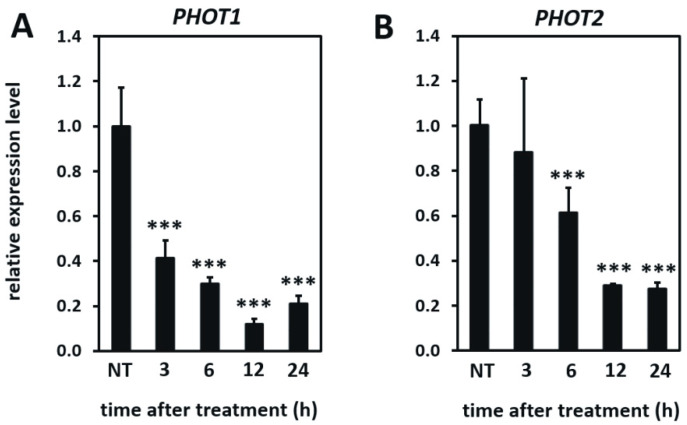
Relative expression level of *PHOT1* (**A**) and *PHOT2* (**B**) genes in the wild-type plants in non-stress conditions and 3, 6, 12, and 24 h after UV-C exposure. Values are means (±SD) from three biological replicates, for which three individual qPCR reactions were performed (*n* = 9). Asterisks indicate statistically significant differences from non-treated plants, at the level *p* < 0.001 (***).

In the next step, we wanted to assess the expression of genes encoding phototropins at the early stages of UV-C induced signaling and cell death onset. The monitoring of both *PHOT1* and *PHOT2* relative expression levels within first 24 h after UV-C stress, using real-time qPCR, indicated that their transcription drops shortly after UV-C treatment (Figure 6). Then, 24 h after UV-C exposure, the expression level of both *PHOT1* and *PHOT2* was four times lower than their expression in non-treated plants. 

Taken together, these results indicate that the photosynthetic processes in phototropin mutants were less affected by UV-C than in the wild-type plants, which is in agreement with the phototropin expression decrease in response to UV-C treatment. On the other hand, our data demonstrate that phototropins participate in the negative regulation of cell death progression.

### 3.5. Differences in the Plant Transcriptome in Photo Mutants in Non-Stress and UV-C Stress Conditions

In order to better understand the molecular mechanisms in phototropin mutants in both non-stress conditions and after UV-C exposure, we monitored gene expression levels by mRNA sequencing (RNA-Seq) analysis.

**Figure 7:**
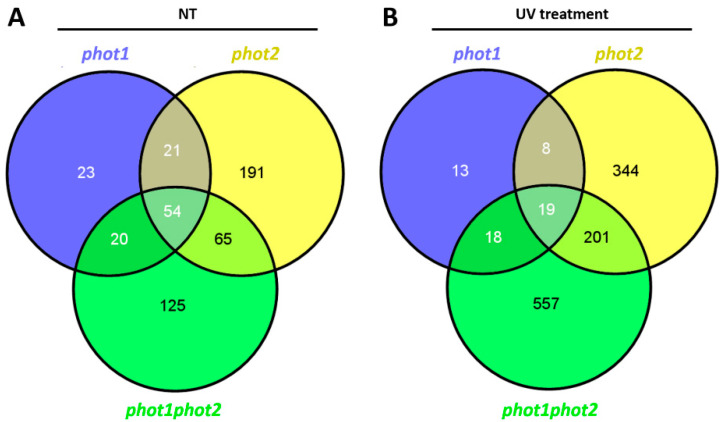
Venn diagram showing numbers of commonly and differentially regulated transcripts in *phot1*, *phot2*, and *phot1phot2* mutants compared to the wild type. (**A**) Numbers of deregulated transcripts in non-stress conditions; (**B**) Numbers of deregulated transcripts 30 min after UV-C exposure.

In non-stress conditions, *phot1*, *phot2*, and *phot1phot2* mutants demonstrated significantly changed expression in 118, 331, and 264 genes, respectively, compared to the wild type (Supplementary Dataset 1). Transcripts with altered expression levels (FDR < 0.01; absolute log2 fold change (FC) > 1) in phototropin mutants were taken for Gene Ontology (GO) term enrichment analysis, which indicated that a more than 20% of differentially expressed genes in each of the *phot* mutants encoded proteins located in the cell periphery, which was defined as the part of a cell encompassing the cell cortex, the plasma membrane, and external encapsulating structures. Functional analysis of differentially expressed genes revealed that they participate in stress response, signaling, transcription regulation, photosynthesis, and cell wall modifications (Supplementary Datasets 2–4). There was a high number of commonly deregulated transcripts when comparing mutants in pairs (Figure 7A). Moreover, under control conditions, 54 genes were differentially regulated in all three tested mutants compared to the wild type (Figure 7A). 

**Figure 8:**
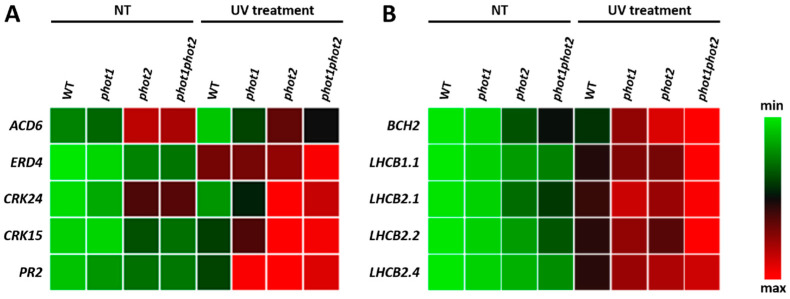
Differentially expressed genes that are potentially involved in phototropin-dependent regulation in photosynthetic reactions (**A**) and signaling (**B**). Color boxes indicate the up-regulation (red) or down-regulation (green). These data are the confirmation of RNAseq results and were performed by qPCR for three additional biological replicates, for which three individual qPCR reactions were performed (*n* = 9).

Interestingly, in all phototropin mutants, we observed an elevated expression level of genes encoding the main chlorophyll a/b-binding proteins of LHCII (LHCB1.1, LHCB2.1, LHCB2.2, LHCB2.4), which was additionally confirmed for another three biological replicates by qPCR analysis (Figure 8A). Moreover, the gene encoding one of the enzymes involved in zeaxanthin synthesis from the β-carotene, *BETA-CAROTENE HYDROXYLASE 2* (AT5G52570), was commonly induced in *phot1* and *phot2* and even higher in *phot1phot2*, in comparison to the wild type. Importantly, we also recognized a couple of membrane proteins involved in signal transduction, such as WALL ASSOCIATED KINASE-LIKE 4 (WAKL, AT1G16150), OXIDATIVE SIGNAL-INDUCIBLE1 (OXI1, AT3G25250), CYSTEINE-RICH RLK (RECEPTOR-LIKE PROTEIN KINASE) 36 (CRK36, AT4G04490), Leucine-rich repeat protein kinase family protein (AT5G37450), and GLUTAMATE RECEPTOR 1.2 (GLR1.2, AT5G48400). They were all deregulated in all *phot1*, *phot2*, and *phot1phot2* mutants, which indicates that they may be involved in phot1- and phot2-dependent signaling. Additionally, only the *phot1phot2* mutant demonstrated the up-regulation of six genes involved in stomata development and functioning. These are genes that are involved in stomata spacing and patterning (*MPK6*–AT2G43790, *STOMAGEN*–AT4G12970) as well as signaling (*CRY1*–AT4G08920, *PHYA*–AT1G09570, *PIF4*–AT2G43010, *BIN2*–AT4G18710) [65]. It seems that these plants try to overcome the influence of *phot* mutations on stomata development by inducing the expression of stomata regulatory genes. 

In a next phase, we considered changes in transcript levels in UV-C treated phototropin mutants versus UV-C treated wild type. In comparison to the wild-type plants, we found 58, 572, and 795 differentially expressed genes (FDR < 0.01; absolute log2 fold change (FC) > 1) in *phot1*, pho*t2*, and *phot1phot2* mutants, respectively (Supplementary Dataset 1) (Figure 7B). Similarly to non-stress conditions, the highest representation of genes deregulated in phototropin mutants encoded the plasma membrane, cell wall, and extracellular region proteins. Moreover, almost 50% of deregulated transcripts in *phot1* and *phot2* and 35% in *phot1phot2* encoded proteins engaged in response to stimuli (Supplementary Datasets 5–7). Functional analysis of differentially regulated transcripts showed that they are engaged in signaling, regulation of transcription, protein modification and degradation, secondary and hormone metabolism. Among genes encoding plasma membrane proteins, we identified many engaged in signal transduction pathways. We selected some of them and additionally confirmed their expression level for another three biological replicates using qPCR (Figure 8B). Among others, there was an up-regulation of *ACCELERATED CELL DEATH 6* (*ACD6*, AT4G14400), encoding a regulator of salicylic acid signaling that shuttles between plasma membrane and cytoplasm to confer stress signal transduction [66]. Moreover, two cysteine-rich RLK (receptor-like protein kinase), CRK15 (AT4G23230), and CRK24 (AT4G23320) were strongly up-regulated in all *phot* mutants. Both these CRKs are involved in ROS sensing [67,68]. Other plasma-membrane bound protein PATHOGENESIS-RELATED PROTEIN 2 (PR2, AT3G57260) and early-responsive to dehydration stress protein (ERD4, AT3G54510), which are also putatively located in chloroplasts, were also up-regulated in phototropin mutants. PR2 was shown to be involved in defense response induction [69]. As a result of the same plasma-membrane localization as the phototropins, these proteins may serve as phot1- and phot2-dependent signaling proteins after UV-C stress. However, to confirm their role in phototropins-dependent signaling, further studies need to be undertaken. Even though there were only 19 genes commonly deregulated in *phot1*, *phot2*, and *phot1phot2* double mutant (Figure 7B), they encode proteins that may have crucial roles in phototropin-dependent response to UV-C. First of all, a gene encoding actin-related protein 9 (ARP9, AT5G43500) [70] demonstrated decreased expression level after UV-C treatment in all tested mutants, compared to the wild type. The specific role of this protein is not known, but it might be engaged in chloroplast-actin filaments reorganization. Moreover, among phototropin-jointly regulated genes after UV-C exposure, we identified some genes, encoding proteins engaged in oxidative stress signaling and response, such as galactinol synthase 2 (GolS2, AT1G56600) and Arabidopsis NAC domain containing protein 29 (AtNAP, ANAC029, AT1G69490), which were upregulated, and downregulated Arabidopsis Tóxicos en Levadura 78 (AtATL78, AT1G49230). GolS2 transcript levels have been shown to rise in response to oxidative damage-inducing agent, and plants over-expressing GolS2 have increased tolerance to salt, chilling, and high-light stress [71,72,73]. ANAC029 is involved in chlorophyll degradation and leaf senescence and functions as a negative regulator in salt stress response [74,75,76], while RING E3 ubiquitin ligase AtATL78 mediates ABA-dependent ROS signaling in response to drought stress [77,78]. They all seem to have an important role in phototropin-dependent signaling during oxidative stress, but in order to elucidate their specific role in these pathways, further studies need to be performed.

Moreover, we found some common genes deregulated in phototropin mutants in our study and in previous transcriptomic analysis of blue-light treated *phot1* and *phot2* [79]. For example, the gene encoding sugar phosphate exchanger (DUF506, AT2G20670), located in the chloroplasts [80], was significantly induced in the *phot1* mutant in both ours and a previous study. We also observed the induction of two genes encoding leaf senescence regulators, *RESPONSIVE TO DESICCATION 26* (*RD26*, AT4G27410) [81,82] and *SENESCENCE-ASSOCIATED GENE 21* (*SAG21*, AT4G02380) in *phot1* and *phot2*, respectively. These genes were previously shown to be upregulated during blue light response in phototropin mutants [79]. After UV-C treatment, we observed the induction of some common genes that were also shown to be upregulated upon blue light treatment in phototropin mutants. These were genes encoding PLAC8 family protein (AT1G52200), which were located on the plasma membrane [83] and engaged in response to oxidative stress [84], high-light induced ETHYLENE RESPONSIVE FACTOR 54 (ERF54, AT4G28140) [85], and chloroplast-located GLUTATHIONE S-TRANSFERASE U17 (GSTU17, AT1G10370) [80].

To sum up, the RNAseq results indicate that genes encoding photosynthetic components are up-regulated in phototropin mutants, both before and after UV-C stress. In both non-stress conditions and after oxidative stress, we also identified many plasma-membrane bound proteins, engaged in signal transduction, that may be involved in phototropin-dependent signaling pathways. However, since there is no indication of the possible phototropin activity as transcriptional regulators, the phototropin-dependent influence on gene expression is most probably indirect.

## 4. Discussion

In this study, we characterized the influence of phototropins on Arabidopsis morphological traits, such as rosette size, dry weight, stomata density, as well as physiological and molecular features, such as water-use efficiency, photosynthetic efficiency, and the transcriptome. The impact of both phot1phot2 double mutations on Arabidopsis leaves curling, observed in this study, has already been described [17,86,87,88,89]. However, the phenotype of *phot* mutants differs depending on light conditions. It was shown by Gotoh and co-workers [41] that *phot2* mutant plants were larger than the wild-type plants and showed an increase in plant biomass in moderate light conditions. Furthermore, recent study analyzing plants engineered to have a slow-photocycling phototropin variant of phot1 displayed increased biomass production as a consequence of their improved sensitivity under low-light and long day conditions [90]. However, in low-light conditions, similar to the intensity of light used in our study, the difference in plant biomass between wild-type and *phot2* mutant plants was rather small [41]. We did not observe any changes in rosette size nor dry weight in single *phot1* and *phot2* mutants, which may be caused by different growing conditions, especially light intensity, used in the current study. However, we detected the biomass reduction in *phot1phot2* mutant, which might be partially connected with leaf flattering and stomata density reduction, but also thinner leaves observed by López-Juez and co-workers [87]. 

Moreover, we showed that apart from the effect on rosette structure, both phot1 and phot2 affect the stomatal density. The number of stomata per mm² of leaf blade was positively influenced by phot1 and phot2 activities. The effect of both phototropins on stomata number seems to be additive, since the *phot1phot2* double mutant demonstrated that stomata density reduced by almost half. The positive influence of phototropins on stomata opening and their additive effect in this process has been already documented [91,92]. However, previous works analyzing stomatal density did not report significant difference between *phot1phot2* and wild-type plants [93,94]. It could be caused by different growing conditions applied, which suggests that the role of phototropins is condition-dependent. Our study indicates that the regulation of transpiration by phototropin is not only through the guard cells operation but also via the condition-dependent regulation of stomata number. The transcriptome profiling performed in this work demonstrated that at least six genes involved in stomata development and patterning were significantly deregulated in the *phot1phot2* mutant, which may indicate that their expression is indirectly regulated by phot1 and phot2 joint activities. However, the specific mechanism of phototropin influence on stomata number is yet to be defined. In our study, lower stomatal density correlated with higher water-use efficiency (WUE) in *phot2* and *phot1phot2* genotypes. These plants used less water for the production of the dry weight unit, which at least partially was connected with reduced transpiration. Even though the stomatal density was reduced in *phot1*, when compared to the wild type, the *phot1* single mutant did not show significant changes in WUE. This may be due to the fact that WUE is a parameter that is influenced by many factors [95] and indicates that the role of phototropins in this process should be further analyzed. Nevertheless, our results confirm previous study showing that the *phot1phot2* double mutant demonstrates reduced stomatal conductance and transpiration [93] and gives additional indication that these physiological traits may be altered by the additive influence of phot1 and phot2 activities on the stomata development. 

Although much research has been done on the role of phot1 and phot2 in the chloroplast movement, so far, relatively little was known about the effect of phot1 and phot2 on PSII efficiency. In this study, we demonstrated that both of them have some impact on maximal and operational PSII efficiency as well as photochemical reactions. The differences in *Fv/Fm*, ΦPSII, and *q*P between wild-type and phototropin mutants were rather small, and thus, they were not the reason for the lower plant biomass in *phot1phot2* under tested growing conditions. Variations in photosynthetic efficiency can be partially explained by the changes in total chlorophyll levels. Previous studies examining photosynthetic parameters did not report significant changes in maximal PSII efficiency between wild-type, *phot1* [42], and *phot2* mutants [41,42,96,97]. Similarly, no statistically significant changes in operational PSII capacity nor photochemical quenching were observed in the *phot2* mutant [96]. It suggests that phototropin-dependent chloroplast movements may be coupled with photosynthetic efficiency in a condition-dependent manner. 

Even though previous works did not demonstrate significant changes in Chl a/b ratio in *phot1* [98] nor *phot2* [41], in the present study, we observed a considerably elevated Chl a/b ratio in the *phot* mutants, which again could be caused by differences in applied growing conditions and indicates the condition-dependent role of phototropins. An increase in the Chl a/b ratio in leaves of phot2-depleted plants may be viewed as a response to higher intracellular light intensity, because the chloroplasts of these plants do not have the avoidance response and thus experience greater photon flux density per PSII in relatively low-light conditions [99]. Such a tendency in the Chl a/b ratio has been demonstrated after exposure to increased irradiance, and it is associated with reduced LHCII size [100]. Moreover, we showed that a lower chlorophyll concentration in the *phot2* and *phot1phot2* mutants correlated with a decreased carotenoids content, which may imply an overall smaller antenna size. However, in order to confirm this, more detailed analyses are needed. Interestingly, in these mutants, we observed elevated transcript levels of genes encoding main chlorophyll a/b-binding proteins of LHCII (LHCB1.1, LHCB2.1, LHCB2.2, LHCB2.4), which may be connected with the efforts of the plants to compensate for a smaller LHCII size. Such a smaller size of light-harvesting antennae may be a photoprotective mechanism in order to avoid photodamage.

Furthermore, the efficiency of NPQ reactions was reduced in all tested mutants (although in *phot2* non-significantly), which suggests that they were able to utilize the energy, absorbed by the antenna, for photochemical reactions rather than for NPQ. These results correlated well with the lower de-epoxidation state of xanthophylls in these genotypes. Although the level of anteraxanthin in all tested genotypes was similar, *phot* mutants differed significantly in the level of zeaxanthin, which was reduced compared to the wild-type plants. Interestingly, the gene-encoding enzyme responsible for zeaxanthin synthesis from the β-carotene, BETA-CAROTENE HYDROXYLASE 2 (AT5G52570), was induced in *phot1* and *phot2* and even higher in *phot1phot2*, in comparison to the wild type. Since the de-epoxidation of violaxanthin, leading to zeaxanthin is decreased in *phot* mutants, they seem to elevate the zeaxanthin level through its synthesis from β-carotene. Recently, a lower NPQ value for the *phot1phot2* mutant was also observed by Howard and co-workers [101].

Since the photosynthetic reactions are tightly connected with oxidative stress and cell death [5,102,103], we wanted to assess the influence of phototropins on the photooxidative stress response. Our results demonstrated that phot1 and phot2 are engaged in the redox regulation in both non-stress and oxidative-stress conditions. We found that H_2_O_2_ levels were significantly decreased in *phot2* and *phot1phot2* plants, which correlated with the lower CAT activity in these mutants. Our results concerning CAT activity differ from the results Kasahara and co-workers of [42], demonstrating no changes in CAT activity in *phot* mutants. The reason for that might be different growing conditions, as we have shown previously that the same mutants may have distant phenotypes depending on ambient conditions [104].

In our previous works, we demonstrated that UV-C radiation, similarly to EEE, causes oxidative stress, destroys photosynthetic apparatus, modifies retrograde signalling from chloroplasts to the nucleus, and causes cell death [2,4,5,9,48,105]. Here, we wanted to uncover the role of phototropins in UV-C-triggered processes. We hypothesized that the mutants impaired with phot2 and thus possessing chloroplasts stacked in the accumulation response will demonstrate a higher level of stress after being exposed to UV-C. Surprisingly, H_2_O_2_ accumulation was significantly diminished in *phot2* and *phot1phot2* mutants in all tested time points after UV-C irradiation compared to the wild type. Additionally, 96 h after UV-C treatment, there was a decrease in H_2_O_2_ content also in the *phot1* mutant, in relation to wild-type, which corresponded with the lower activity of SOD and partially also CAT. The activity of APX showed to have higher dynamics than the other antioxidant enzymes and fluctuated significantly in the course of measurements. 

Generally, the chlorophyll *a* fluorescence measurements performed for UV-C treated plants showed that the photosynthetic machinery was less affected in plants depleted with phototropins than in the wild type. Both maximum and operational photosynthetic efficiency were higher after UV-C irradiation in all phototropin mutants. In addition, their vitality was elevated when compared to Col-0. Since the damages within the photosynthetic machinery seem to be smaller in *phot* mutants, compared to the wild type, they were able to more efficiently use energy harvested by LHCs for photochemical reactions, which could lead to lower H_2_O_2_ over-production, especially in *phot2* and *phot1phot2* mutants. Our results suggest that phot1 and phot2 may be directly or indirectly involved in the adjustment mechanism, such as antenna size optimization, in order to avoid the damaging effects of UV-C.

Importantly, both *phot1* and *phot2* mutants demonstrated higher NPQ, which means that they more efficiently dissipated light energy as heat, which is one of the chloroplast protection mechanisms. These results are in agreement with the decreasing expression level of both *PHOT1* and *PHOT2* genes, and they suggest that plants deliberately decrease phot1 and phot2 levels in order to diminish negative changes caused by stress factor. The reduction in *PHOT2* expression level was also observed after the inhibition of photosynthesis with 3-(3,4-dichlorophenyl)-1,1-dimethylurea (DCMU) [106]. These results and data obtained here suggest that the chloroplast NPQ- and the plastoquinone redox status-dependent retrograde signaling from photoinhibited chloroplasts causes the decline in *PHOT2* gene transcription in order to reduce avoidance response and harvest more light from still operating LHCs and reactions centers. However, this hypothesis needs to be further validated.

There is growing evidence that phot1 and phot2 participate in plant responses to different stresses. For instance, phot1 enhances plant fitness and performance under drought [107], and both phot1 and phot2 are required to recover from high light stress [101,108]. Even though phototropin mutants, especially *phot2* and *phot1phot2*, showed higher photosynthetic electron transport and elevated H_2_O_2_ to significantly lower values, compared to the wild type, it did not compensate their lower stomatal conductance, probably high level of photorespiration, and elevated cell death. These results indicate that despite some protective mechanisms against adverse conditions, such as the protection of photosystems and keeping a relatively low content of H_2_O_2_, *phot* mutants are more prone to cell death, which is most probably because of their disturbed ability in chloroplasts movements. Similar results were shown for *phot2* and *phot1phot2* mutants in response to high light stress [101,108]. In addition, some differences among *phot1* and *phot2* mutants, such as the transcriptomic changes observed in this work, may be connected with different amounts of phototropins on the chloroplasts outer membrane, that in fact may reflect differences in accumulation/avoidance response and may have consequences on many cellular pathways [36]. We hypothesize that phototropin mutants, due to the loss of chloroplast movement ability and reduced stomatal conductance, needed to lower H_2_O_2_ levels, which in turn caused deregulation in electron transport in PSII, increased cell death, and thus reduced growth. However, to confirm this scenario, further studies need to be performed.

The analysis of transcriptomic changes indicated that a great proportion of deregulated transcripts in *phot* mutants encoded proteins associated with biological membranes. Plasma membrane, Golgi/post Golgi vesicles, and chloroplast outer membrane are the main subcellular compartments of phot1 and phot2 localization and phototropin-mediated signaling [36,39,109]. Thus, it seems that phototropins indirectly regulate the expression level of membrane-bound proteins that may be involved in phototropin-dependent signaling pathways. There is a growing evidence that phototropin-mediated asymmetric growth processes are directly linked with auxin and auxin influx and efflux carriers [110]. However, this needs to be regulated rather post-transcriptionally, because we did not observe any differences in the expression of auxin carriers. The only gene involved in auxin metabolism/signaling that was significantly induced in all *phot* mutants was *AT3G44300 encoding nitrilase 2* (*NIT2*), which converts indole-3-acetonitrile to indole-3-acetic acid.

## 5. Conclusions

In conclusion, our findings indicate that phototropins are engaged in the condition-dependent regulation of plant biomass. Both phot1 and phot2 positively influence the stomata density and thus negatively regulate WUE. Moreover, we demonstrated the novel role of both phot1 and phot2 in the regulation of photosynthetic pigments composition, PSII efficiency, and redox status both under non-stress and oxidative stress conditions. Our findings shed new light on the possible signaling pathways that involve phototropins, which can be further studied to holistically understand the role of these photoreceptors in plant cells.

## Figures and Tables

**Table 1:** Chlorophylls and carotenoids contents in 4-week-old wild type and mutant plants.

	WT	*phot1*	*phot2*	*phot1phot2*
**Total Chlorophyll**	28680 ± 2440	29378 ± 2175	25300 ± 1876 **	26628 ± 2298 *
**Chlorophyll *a***	20739 ± 1752	21496 ± 1599	18469 ± 1382 **	19626 ± 1693 *
**Chlorophyll *b***	7941 ± 688	7882 ± 583	6830 ± 508 ***	7002 ± 605 **
**Chlorophyll *a/b***	2.61 ± 0.01	2.73 ± 0.04 ***	2.70 ± 0.06 ***	2.80 ± 0.004 ***
**Lutein**	20575 ± 1592	19440 ± 1445 *	17740 ± 1397 ***	18028 ± 1413 ***
**(A/2 + Z)/(V + A + Z)**	0.081 ± 0.002	0.057 ± 0.005 ***	0.071 ± 0.002 ***	0.074 ± 0.004 ***
**β-Carotene**	5515 ± 480	5353 ± 396	5176 ± 455	4635 ± 408 ***

Total chlorophyll, chlorophyll *a*, chlorophyll *b*, chlorophyll *a/b* ratio; lutein; de-epoxidation state of xanthophyll cycle carotenoids calculated as (A/2 + Z)/(Z + A + V); (Z, zeaxanthin; V, violaxanthin; A, antheraxanthin), and *β-*carotene. Values are means (±SD) of 9−12 plants per genotype from at least two independent experiments (*n* = 9–12) expressed as the peak area per µg of dry weight. Asterisks indicate significant differences from the wild type according to the Tukey HSD test at the level of *p* < 0.05 (*), *p* < 0.005 (**), or *p* < 0.001 (***).

## Data Availability

The data presented in this study are available in supplementary material here at www.mdpi.com/xxx/s1.

## Supplementary Materials

The following are available online at https://www.mdpi.com/2073-4409/10/2/200/s1, Figure S1: Rosette morphology of 4-week-old *Arabidopsis thaliana* wild-type (WT) and phototropin mutants (*phot1*, *phot2*, and *phot1phot2*), in the Col-gl1 background. Pictures were taken for plants 24, 48, and 96 h after UV-C exposure (100 mJ cm^−2^), Table S1: List of genes and their corresponding primers, used for qPCR, Datasets S1–7: Data gathered for analyses.
