# Supplementary material for: Phototropin 1 and 2 Influence Photosynthesis, UV-C Induced Photooxidative Stress Responses, and Cell Death
Source: Cells. 2021 Jan 20;10(2):200. doi: 10.3390/cells10020200 (PMC7909289; doi:10.3390/cells10020200)
Supplement: Supplementary file 1 [file cells-10-00200-s001.zip › cells-1039510 supplementary proofreading back - Copy/Rusaczonek et all_Supplementary Material_19.01.2021.pdf]

Supplementary Data

Phototropin 1 and 2 Influence Photosynthesis, UV-C Induced Photooxidative Stress Responses, and Cell Death

Anna Rusaczonek [1,2,*], Weronika Czarnocka [1,2], Patrick Willems [3,4], Marzena Sujkowska-Rybkowska [1], Frank Van Breusegem [3,4] and Stanisław Karpiński [2,*]

1 Department of Botany, Institute of Biology, Warsaw University of Life Sciences, Nowoursynowska 159, 02-776 Warsaw, Poland; weronika_czarnocka@sggw.edu.pl (W.C.); marzena_sujkowska@sggw.edu.pl (M.S.-R.)
2 Department of Plant Genetics, Breeding and Biotechnology, Institute of Biology, Warsaw University of Life Sciences, Nowoursynowska 159, 02-776 Warsaw, Poland
3 Department of Plant Biotechnology and Bioinformatics, Ghent University, Technologiepark 71, 9052 Ghent, Belgium; patrick.willems@psb.vib-ugent.be (P.W.); frank.vanbreusegem@psb.vib-ugent.be (F.V.B.)
4 VIB Center of Plant Systems Biology, Technologiepark 71, 9052 Ghent, Belgium
* Correspondence: anna_rusaczonek@sggw.edu.pl (A.R.); stanislaw_karpinski@sggw.edu.pl (S.K.)

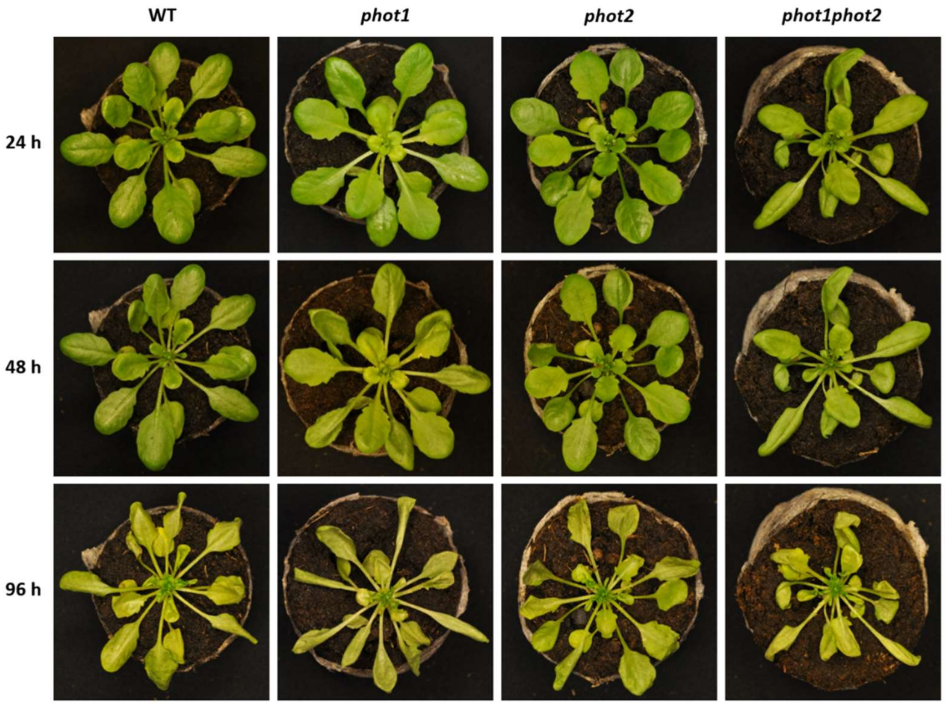

Figure S1. Rosette morphology of 4-week-old *Arabidopsis thaliana* wild-type (WT) and phototropin mutants (*phot1*, *phot2*, and *phot1phot2*), in the Col-gl1 background. Pictures were taken for plants 24, 48 and 96 h after UV-C exposure (100 mJ cm^{-2}).

Table S1. List of genes and their corresponding primers, used for qPCR.

Gene abbreviation	Gene name	AGI	Forward primer	Reverse primer
5-FCL	*5-FORMYLTETRAHYDROFOLATE CYCLOLIGASE*	AT5G13050	GCAAACTCAATGAACATTTTGG	GATCGGTTCATCTGCTTGC
ACD6	*ACCELERATED CELL DEATH 6*	AT4G14400	GGGACGAACCTTAAGCAAAA	CAGAACTGTCCATCGCAAAC
BCH2	*BETA CAROTENOID HYDROXYLASE 2*	AT5G52570	GGCTATAATGGCTGTTTATTACCG	ACATCTCTAACACAGACACTTCACCT
CRK15	*CYSTEINE-RICH RLK (RECEPTOR-LIKE PROTEIN KINASE) 15*	AT4G23230	CACCACCGCTACCTCCTTC	CACATTTGAATTCCAACTTTTCC
CRK24	*CYSTEINE-RICH RLK (RECEPTOR-LIKE PROTEIN KINASE) 24*	AT4G23320	AATATTCGTCCTCGTCAAAAAGA	GAACAACGATAATCGCCACA
ERD4	*Early-responsive to dehydration stress protein*	AT3G54510	CCAGGGAATTGCAAAGTATGA	CAACTGAGCTAAGCCCTGGTA
LHCB1.1	*LIGHT HARVESTING CHLOROPHYLL A/B-BINDING PROTEIN 1.1*	AT1G29920	TGCGGCATCAGAAGTCCT	TGCCTGATGGACCCTTTG
LHCB2.1	*LIGHT HARVESTING CHLOROPHYLL A/B-BINDING PROTEIN 2.1*	AT2G05100	AGTCTACTCCTCAAAGCATCTGGT	CGGTTAGGTAGGACGGTGTATT
LHCB2.2	*LIGHT HARVESTING CHLOROPHYLL A/B-BINDING PROTEIN 2.2*	AT2G05070	TACTCCCCAAAGCATCTGGT	CCGGGGTATTCTCCAGTTAAG
LHCB2.4	*LIGHT HARVESTING CHLOROPHYLL A/B-BINDING PROTEIN 2.4*	AT3G27690	GCGTCGTACCGTCAAGTCTAC	GGAAAATGGTCCTAGGTATTTGG
PHOT1	*PHOTOTROPIN 1*	AT3G45780	CAACGAAATTCCCGAGAAAA	TCGTCTATAGATTCATCCAAAGATTC
PHOT2	*PHOTOTROPIN 2*	AT5G58140	GCTACCACTCTTGAGCGCATA	CAAGAAAGCTGTCTGATGCAAA
PP2AA2	*PROTEIN PHOSPHATASE 2A SUBUNIT A2*	AT3G25800	TGCAATGGTTACAAGACAAGGTT	GCATTGCCCATTCAGGAC
PR2	*PATHOGENESIS-RELATED PROTEIN 2*	AT3G57260	GCTTAGCCTCACCACCAATG	CCCGTAGCATACTCCGATTT
YLS8	*YELLOW-LEAF-SPECIFIC GENE 8*	AT5G08290	TTACTGTTTCGGTTGTTCTCCATTT	CACTGAATCATGTTCGAAGCAAGT
